# Genomics in 2012: challenges and opportunities in the next generation sequencing era

**DOI:** 10.1186/1471-2164-13-S8-S1

**Published:** 2012-12-17

**Authors:** Zhongming Zhao, Yufei Huang, Bing Zhang, Yu Shyr, Hua Xu

**Affiliations:** 1 Department of Biomedical Informatics, Vanderbilt University School of Medicine, Nashville, TN 37232, USA; 2 Department of Psychiatry, Vanderbilt University School of Medicine, Nashville, TN 37232, USA; 3 Department of Cancer Biology, Vanderbilt University School of Medicine, Nashville, TN 37232, USA; 4 Center for Quantitative Sciences, Vanderbilt University, Nashville, TN, USA; 5 Department of Electrical and Computer Engineering, The University of Texas at San Antonio, San Antonio, TX, USA; 6 Department of Biostatistics, Vanderbilt University School of Medicine, Nashville, TN, USA

## Abstract

We present a report of the 2012 International Conference on Intelligent Biology and Medicine (ICIBM 2012) and the editorial report of the supplement to *BMC Genomics *that includes 22 research papers selected from ICIBM 2012, which was held on April 22-24, 2012 in Nashville, Tennessee, USA. The conference covered a variety of research areas, including bioinformatics, systems biology, and intelligent computing. It included six sessions, a tutorial - Introduction to Proteome Informatics, a workshop - Next Generation Sequencing, and a poster session. The selected papers in this Supplement issue represent the genomic focus in ICIBM 2012.

## Introduction

The past decade has witnessed rapid development in high-throughput technologies that are capable of uncovering millions of genetic variants, genome-wide transcription differences among individuals at both the mRNA and microRNA levels, genome-wide methylation status at high resolution, structural variants such as copy number gains or losses, proteomics and metabolomics information, and bioimaging, among others [1]. Recently, massively parallel sequencing, commonly called next generation sequencing (NGS), has been rapidly transforming biological and biomedical research. Massive amounts of high-dimensional data have provided us new opportunities to advance biological and biomedical research; however, there have been substantial challenges [2-4] in the storage, analysis, integration and reduction of those cross-platform, multi-dimensional, heterogeneous data in order to identify true biological signals, biomarkers and disease susceptibility genes. Systems biology approaches to investigate the dynamic and complicated interactions and regulations in biological and cellular systems are much needed. Recently, there have been numerous successful systems biology applications, though we have also seen strong limitations such as incomplete and erroneous data, lack of robust algorithms or methods, and insufficient knowledgebase. For example, the canonical pathways from the popular KEGG pathway database contain only one-third of human protein-coding genes [4,5]. Furthermore, genomic analysis has been applied to many other disciplines such as pharmacology, clinical decision support and research, biophysics, and nanotechnologies. One key issue in genomics and systems biology is to develop an effective and efficient method to extract useful information from the specific data.

To bridge the gaps in biological and biomedical research at different levels of study related to living organisms, to foster interdisciplinary and multidisciplinary research, and to provide education and training opportunities to students and investigators in life sciences, medicine, computer science, bioengineering, statistics, mathematics, and biophysical sciences, we organized the 2012 International Conference on Intelligent Biology and Medicine (ICIBM 2012), held in Nashville, Tennessee, USA, April 22-24, 2012 [6]. The conference covered three areas: bioinformatics, systems biology, and intelligent computing. This supplement issue is devoted to ICIBM 2012 with the focus on genomics, especially NGS technologies and applications.

## ICIBM 2012 scientific program

The scientific program includes three keynote speakers who are world renowned leaders in genomics, systems biology, biomedical informatics, and translational sciences, six scientific sessions, one poster session, one tutorial, and one workshop. In addition to the poster session, which hosted 23 abstract-based presentations, the conference organizers also hosted a reception in the first day to promote interactions and discussion among all attendees. This reception was well received, as numerous junior researchers had ample opportunities to directly interact with senior investigators in a multidisciplinary fashion. Moreover, we were able to provide 15 travel awards to trainees across the United States, and even one international scholar, thanks to grant support from National Science Foundation. The travel awards were selected by the Award Committee from a substantial number of outstanding manuscripts and abstracts that spanned the wide variety of our research subjects.

In the following, we first briefly review the keynote speakers' lectures and then the regular sessions, followed by the workshop and tutorial.

Three keynote speakers delivered talks on their cutting-edge research and shared their views and perspectives of their research fields. These speakers were Dr. Wen-Hsiung Li from the University of Chicago, Dr. Randolph A. Miller from Vanderbilt University, and Dr. Brian D. Athey from the University of Michigan.

"*Protein Structure, Function and Classification*" In this keynote lecture, Dr. Wen-Hsiung Li presented his recent work in the investigation of the relationship between protein structure and function, focusing on the functional surfaces of protein. Based on the available ~30,000 functional surfaces of bound forms of ~68,000 structures annotated by Protein Data Bank (PDB), the new method developed in Dr. Li's lab could effectively perform protein functional surface classification, which could be particularly useful for detecting evolutionary relationships among divergent sequences because functional surfaces tend to be well conserved in evolution. Medical applications were also discussed in this talk. Dr. Wen-Hsiung Li is the James D. Watson Chair Professor at the University of Chicago and a member of the National Academy of Sciences, USA. He is best known for his studies on the molecular clock (i.e., rates and patterns of DNA sequence evolution) and on the patterns and consequences of gene duplication. His research interests include evolutionary genomics, molecular evolution, bioinformatics and computational biology, population genetics, and human genetics.

"*Introducing tranSMART: An Open Source and Community-driven Data Sharing and Analytics Platform for Translational Research*" Dr. Brian D. Athey reviewed the current trends in biomedical computing and then introduced tranSMART, a next-generation analytical and data sharing informatics platform for translation research. Dr. Athey provided his vision for enhancing tranSMART that leverages NIH-funded projects (i2b2, NCIBI, and NCBO) and emerging public-private partnerships that scale locally, regionally, nationally, and internationally. Dr. Athey is the Collegiate Professor and Inaugural Chair of the Department of Computational Medicine and Bioinformatics at the University of Michigan. He is the founding Principal Investigator of the NIH National Center for Integrative Biomedical Informatics (NCIBI), one of eight NIH National Biomedical Computing Centers. He also serves as the Biomedical Informatics Director of Michigan's Clinical and Translational Science Award (CTSA), and is the Associate Director of the Michigan Institute for Clinical and Health Research (MICHR).

*"A General Introduction to the Art and Practice of Clinical Decision Support" *In this keynote lecture, Randolph A. Miller, a pioneer in clinical decision support (CDS), provided a comprehensive review of the history and growth of CDS and future opportunities and challenges. Dr. Miller is the Cornelius Vanderbilt Professor of Biomedical Informatics and a University Professor of Biomedical Informatics, Medicine, and Nursing. He is a member of the Institute of Medicine (IOM) of the National Academy of Sciences, the founding Chair of the Department of Biomedical Informatics (DBMI) at Vanderbilt University, and the President of the American College of Medical Informatics (2003-2004).

ICIBM 2012 had six regular scientific sessions for researchers to showcase their recent innovative work in the areas of bioinformatics, systems biology, medical informatics, and intelligent computing. The presenters were chosen through a rigorous review process, and their work stood out among the submissions as novel and significant. These sessions are

Session I: Genomics

Session II: Systems biology I

Session III: Algorithms and methods

Session IV: Intelligent computing

Session V: Applications and tools

Session VI: Systems biology II.

We do not provide much detail of these sessions here because most of the talks were selected from the high-quality submitted papers, and details of the work can be found in the introduction of each supplement issue as well as the papers. Additionally, the details of each session, including session chairs, speakers, and title and abstract of each talk, are available online [6] and in the conference program book.

Finally, ICIBM 2012 included a workshop and a tutorial for educational purposes, both of which attracted many attendees and were very well received.

"*Workshop on next-generation sequencing*" This workshop was organized by Dr. Kun Huang from The Ohio State University and Dr. Dongxiao Zhu from Wayne State University. NGS is rapidly emerging as a powerful high throughput genomic approach in biomedical and biological research. The large volume of data and complicated data analysis pipelines has brought us a significant challenge in both technology development and practical applications. The goal of this workshop is to bring together the NGS researchers and others of interest to introduce the cutting edge technologies, report their recent research results, share new ideas, discuss current challenges and future opportunities, and network among attendees. This workshop had two sessions. *Session I: RNA-seq data analysis *focused on whole transcriptome sequencing including the presentations of three tools: *Alt Event Finder *for alternative splicing event detection, *SASeq *for detecting and quantifying active transcripts, and *DFI *for gene feature discovery in RNA-seq experiments from multiple sources. *Session II: NGS data sharing and systems biology *covered NGS data sharing practice in Vanderbilt University, regulatory network analysis based on a Bayesian algorithm, and methylCap-seq developed in a real DNA methylation project taking advantage of NGS technologies.

"*Tutorial: introduction to proteome informatics*" This tutorial was offered by Dr. David L. Tabb at Vanderbilt University and had four instructors: Drs. David L. Tabb, Bing Zhang, Qi Liu, and Xiaojing Wang. During the last decade, numerous algorithms have been developed for tandem mass spectrometry data. Currently, identifying proteins and post-translational modifications (PTMs) from tandem mass spectrometry data heavily relies on specific algorithms. This tutorial introduces major elements of the protein and PTM identification pipeline and describes strategies for comparative proteomics. The second half describes techniques to address biological questions through integrating proteomic data with genomic data. The content is designed to be accessible to computer scientists and bioinformaticians who have not previously worked with proteomics data sets.

## *BMC Genomics *supplement issue

This supplement issue included 22 original papers selected from the submissions to the ICIBM 2012, after rigorous peer review, reflecting current hot research areas (NGS, methylation genomics, microRNA regulation, whole transcriptome sequencing, etc.) and the broadness in genomic research (genomic data analysis, algorithms, bioinformatics software, data mining, biomedical applications, etc.). We attempted to order these papers logically, even though the topics are quite diverse.

Xu et al., [7] reported an investigation of gene expression in schizophrenia, a heritable complex mental disorder, using RNA sequencing (RNA-Seq) technology. They found 198 genes differentially expressed between cases and controls, 21 of which reached nominal significance in gene-based association analyses of a genome-wide association dataset. Pathway analysis revealed that these genes were highly enriched in immune related pathways, implicating the involvement of immune system in schizophrenia. Based on the evaluation and comparison of the statistical approaches for analyzing high-throughput RNA interference (RNAi) screening simulation data, Ye et al., [8] assessed promising methods using real data from a loss-of-function RNAi screen to identify hits that modulate paclitaxel sensitivity in breast cancer cells. They identified a number of gene targets with inhibitors known to enhance paclitaxel sensitivity, suggesting other identified genes may merit further investigation. Harwich et al., [9] reported the genomic sequence analysis and characterization of a novel species, *Sneathia amnii *sp. nov, which closely resembles bacteria previously designated "*Leptotrichia amnionii*." This study is part of the Vaginal Human Microbiome Project conducted at the Virginia Commonwealth University.

Histone modification plays an important role in cell differentiation and tissue development in eukaryotes. Zhang et al., [10] applied several clustering methods including K-means, hierarchical and principle component analysis on the dimethylation of lysine 4 residue on histone 3 (H3K4me2) ChIP-seq data from embryonic stem cells, neural progenitor cells and whole brains of mice, aiming to identify genes with the H3K4me2 binding on the gene body region in different cell development stages and study their redistribution in different tissue development stages. DNA methylation is an important epigenetic regulation in the cellular system. Genome-wide methylation profiling has been growing quickly but many challenges exist. Trimarchi et al., [11] presented their optimal approach to reduce noise and enhance the identification of methylation events from the MethyCap-seq data using NGS. Xia et al., [12] conducted a comprehensive analysis of the mutation rate of methylated cytosines from human embryonic stem cells using genome-wide single-base resolution methylation data. They found a high mutation rate in low-intermediately to intermediately methylated CpG sites and a significant correlation between the methylation level and cytosine allele frequency. Their findings provide the first supporting evidence of mutation rate variation at human methylated CpG sites using the genome-wide sing-base resolution methylation data.

Novel algorithms, approaches and methods, and computational tools are much needed in genomic studies. Liu et al., [13] performed a systematic evaluation of each step in three popular tools (SAMtools, GATK, and GlfMultiples) for SNP and genotype calling using NGS data. Yuan et al., [14] presented a stand-alone, efficient, and user-friendly software tool, BM-Map, for accurately allocating multireads from RNA-seq data. The internal algorithm is based on a Bayesian stochastic model to calculate mapping probabilities of multireads for competing genomic loci. Zhou et al., [15] introduced Alt Event Finder, a tool for identifying novel splicing events by using transcript annotation derived from genome-guided construction tools, such as Cufflinks and Scripture. Another RNA-seq based tool, DFI, was developed by Ozer et al., [16] for accurate identification of gene expression changes in multiple RNA-seq datasets. The tool is built on a non-parametric and unsupervised method using a metric called Differential Feature Index (DFI). In addition to RNA-seq based tools, Schweikert et al., [17] reported a combinatorial fusion strategy that using score or rank could improve the peak detection of ChIP-seq data.

MicroRNAs are short (19-25 nucleotides), non-coding RNAs playing a key function in post-transcriptional regulation. Three papers in this issue are related to microRNA bioinformatics. Yue et al., [18] reported an algorithm called BCmicrO that combines the algorithms for microRNA target prediction with Bayesian Network (BN). The authors showed that BCmicrO could outperform each individual algorithm measured by both sensitivity and specificity. Zeng et al., [19] demonstrated an integrative approach to studying differential combinatorial regulatory networks in venous metastasis of hepatocellular carcinoma. Both the microRNA and transcription factor regulations are integrated in their network analysis. Finally, Wan et al., [20] proposed a novel computational method for microRNA family detection. The method employs *n*-gram to transform primary sequences to numeric vectors and uses *k*-means to automatically cluster these vectors.

With numerous genetic and genomic datasets available for a complex disease, it is important to apply integrative approaches to ranking candidate genes for future validation. Zhao et al., [21] applied a unique multi-species evidence-based data integration strategy using genetic and genomic datasets for alcoholism from four species (humans, mice, *C. elegans *and *Drosophila*). They developed permutation and false discovery rate (FDR) strategies to find an optimal weighting matrix and to evaluate the ranking results using a genome-wide association studies dataset. Their functional analyses of the top ranked genes suggested the approach is useful to identify candidate genes contributing to alcoholism. Fettweis et al., [22] constructed the Vaginal 16S rDNA Reference Database, a comprehensive and non-redundant database of 16S rDNA reference sequences for bacterial taxa likely to be associated with vaginal health. They further developed STIRRUPS, a new method that employs the USEARCH algorithm with a curated reference database for rapid species-level classification of 16S rDNA partial sequences. He et al., [23] presented CTF, a novel integrated transcription factor binding site (TFBS) prediction system based on Conditional Random Fields (CRFs) framework. Going from TFBS to another important sequence feature in the genome, Deng et al., [24] applied a new recursive entropic segmentation method and nucleotide doublets statistics to detect the borders between coding and non-coding DNA regions in prokaryotes.

The last four papers in this issue are more related to genomic and biomedical data mining. Jourquin et al. [25] present GLAD4U, a freely available web-application for creating expert candidate gene lists tailored to a user's query. GLAD4U ensures computational efficiency through the effective use of existing NCBI resources, which also made it one of the winning applications in the National Library of Medicine (NLM)'s 2011 Software Development Challenge on the Innovative Uses of NLM Information. Wu et al., [26] developed a two-stage classification method for identifying genes and genetic lesion statuses in clinical trial documents. Their system was initially developed and tested on individually annotated genes and later was expanded to all genes in cancer trial documents. The gene-neutral classifier achieved a highest accuracy of 89.8%, indicating the system's potential in facilitating information retrieval tasks targeting clinical trial documents. Statnikov et al., [27] used transcription factor-target gene regulatory interactions to evaluate a new family of methods that, given observational data for just two causally related variables, can determine which is the cause and which is the effect. The authors also introduced a novel ensemble technique for causal orientation that combines decisions of individual methods. The ensemble method was found to be more accurate than any individual causal orientation method. In the last paper, Xu et al., [28] presented their new methods based on literature cohesion to objectively evaluate the overall functional significance of gene expression experiments, compare different statistical methods, and determine the appropriate statistical P-value threshold. Their approach provides an objective biological metric to filter the vast amount of publicly available microarray experiments for subsequent meta-analysis and systems biology research.

## Conference organization

### 2012 International Conference on Intelligent Biology and Medicine (ICIBM 2012) (April 22-24, 2012, Nashville, Tennessee, USA)

Our sincerest thanks to the members of our Steering, Program, Award, Publicity, and Local Organization committees, as well as our numerous reviewers and volunteers, for the countless hours and energy spent to make ICIBM 2012 a success! We could not have accomplished so much without the dedication of each and every person that contributed to this conference.

**Sponsors **National Science Foundation, Vanderbilt University (VU), Vanderbilt Center for Quantitative Sciences, Bioinformatics Resource Center at Vanderbilt-Ingram Cancer Center, International Society of Intelligent Biological Medicine, Shanghai Center for Bioinformation Technology, China, and Shanghai Jiao tong University, China.

**Partners **Vanderbilt University (Center for Quantitative Sciences, Bioinformatics Resource Center), Meharry Medical College, and Tennessee State University.

**General Chairs **Zhongming Zhao (Vanderbilt University) and Yu Shyr (Vanderbilt University).

**Steering Committee **Chair: Zhongming Zhao (Vanderbilt University). Members: A. Keith Dunker, Indiana University, Yixue Li (Shanghai Center for Bioinformation Technology, China), Dong Xu (University of Missouri- Columbia), and Ying Xu (University of Georgia).

**Program Committee **Chairs: Hua Xu (Vanderbilt University) and Yufei Huang (University of Texas-San Antonio). Members: William S. Bush (Vanderbilt University), Jake Chen (Purdue University), Liz Chen (University of Vermont-Burlington), Xue-Wen Chen (The University of Kansas), Juan Cui (University of Georgia), Qinghua Cui (Peking University, China), Youping Deng (Rush University Medical Center), Joshua Denny (Vanderbilt University), Marcelo Fiszman (National Library of Medicine, National Institutes of Health), Jan Freudenberg (Feinstein Medical Research Institute), Ge Gao (Peking University, China), Mark Gerstein (Yale University), Chittibabu (Babu) Guda (University of Nebraska Medical Center), Ramin Homayouni (University of Memphis), Steve Horvath (University of California-Los Angeles), Xiaohua Tony Hu (Drexel University), Weichun Huang (National Institute of Environmental Health Sciences), Yang Huang (Kaiser Permanente), Jenn-Kang Hwang (National Chiao Tung University), Peilin Jia (Vanderbilt University), Yufang Jin (University of Texas-San Antonio), Sun Kim (Seoul National University, Korea), Judith Klein-Seetharaman (University of Pittsburgh), Dmitry Korkin (University of Missouri-Columbia), Leping Li (National Institute of Environmental Health Sciences), Liao Li (University of Delaware), Honghuang Lin (Boston University), Chunyu Liu (University of Chicago), Hongfang Liu (Georgetown University), Tianming Liu (University of Georgia), Yulong Liu (Indiana University), Patricio A Manque (Universidad Mayor, Chile), Xizeng Mao (University of Georgia), Ranadip Pal (Texas Tech University), Yi Pan (Georgia State University), Yonghong Peng (University of Bradford, UK), Jiang Qian (Johns Hopkins University), Thomas Rindflesch (National Institutes of Health), Marylyn Ritchie (Penn State University), Neil Sarkar (University of Vermont, Burlington), Alexander Statnikov (New York University Langone Medical Center), Jingchun Sun (Vanderbilt University), Manabu Torii (Georgetown University Medical Center), Jun Wan (John Hopkins University), Yufeng Wang (University of Texas at San Antonio), Qingguo Wang (Vanderbilt University), Xiaoyan Wang (University of Connecticut), Yonghui Wu (Vanderbilt University), Lu Xie (Shanghai Center for Bioinformation Technology), Jianhua Xuan (Virginia Tech), Bing Zhang (Vanderbilt University), Yanqing Zhang (Georgia State University), Min Zhao (Vanderbilt University), Huiru (Jane) Zheng (University of Ulster, UK), and W. Jim Zheng (Medical University of South Carolina).

**Award Committee **Chair: Bing Zhang (Vanderbilt University). Members: Siddharth Pratap (Meharry Medical College), Sachin Shetty (Tennessee State University), and Dave Tabb (Vanderbilt University).

**Publicity Committee **Chairs: Lu Xie (Shanghai Center for Bioinformation Technology, China) and Yonghong Peng (University of Bradford, UK)

**Local Organization Committee **Chair: Rebecca Hiller Posey (Vanderbilt University). Members: Jillanne K. Shell (Vanderbilt University), Michael Guo (Vanderbilt University), Qi Liu (Vanderbilt University), Jingchun Sun (Vanderbilt University), Qingguo Wang (Vanderbilt University), Yonghui Wu (Vanderbilt University), and Min Zhao (Vanderbilt University).

## Competing interests

The authors declare that they have no competing interests.
